# Roaming Dynamics and Conformational Memory in Photolysis of Formic Acid at 193 nm Using Time-resolved Fourier-transform Infrared Emission Spectroscopy

**DOI:** 10.1038/s41598-020-61642-7

**Published:** 2020-03-16

**Authors:** Cheng-Jui Tso, Toshio Kasai, King-Chuen Lin

**Affiliations:** 1 Department of Chemistry, National Taiwan University, Taipei, 10617 Taiwan; 2 Institute of Atomic and Molecular Sciences, Academia Sinica, Taipei, 10617 Taiwan; 3 Institute of Scientific and industrial Research, Osaka University, Ibaraki, Osaka 567-0047 Japan

**Keywords:** Photochemistry, Physical chemistry

## Abstract

In photodissociation of trans-formic acid (HCOOH) at 193 nm, we have observed two molecular channels of CO + H_2_O and CO_2_ + H_2_ by using 1 μs-resolved Fourier-transform infrared emission spectroscopy. With the aid of spectral simulation, the CO spectra are rotationally resolved for each vibrational state (v = 1–8). Each of the resulting vibrational and rotational population distributions is characteristic of two Boltzmann profiles with different temperatures, originating from either transition state pathway or OH-roaming to form the same CO + H_2_O products. The H_2_O roaming co-product is also spectrally simulated to understand the interplay with the CO product in the internal energy partitioning. Accordingly, this work has evaluated the internal energy disposal for the CO and H_2_O roaming products; especially the vibrational-state dependence of the roaming signature is reported for the first time. Further, given a 1 μs resolution, the temporal dependence of the CO/CO_2_ product ratio at v ≥ 1 rises from 3 to 10 of study, thereby characterizing the effect of conformational memory and well reconciling with the disputed results reported previously between absorption and emission methods.

## Introduction

Roaming is recognized as one of the hottest topics in reaction dynamics for the past decade. For the most studied roaming cases of H_2_CO and CH_3_CHO^1–11^, following photodissociation a recoiling atom or radical fragment on the ground-state surface can roam and then abstract intramolecularly another H atom to generate the H_2_ + CO and CH_4_ + CO molecular products. This pathway is expected to open within the roaming window, in which the dissociation threshold of such a radical channel and the transition state (TS) of a molecular channel are close to each other. However, the roaming dynamics turns out to be more complicated to render studies oriented in varied directions^12–25^. As an example using carbonyl compounds in the photodissociation, aliphatic aldehydes showed a loose roaming saddle point along the roaming dissociation coordinate^26,27^, whereas the roaming signature in methyl formate (HCOOCH_3_) was verified to involve multiple energy states via a conical intersection^17,18^. These two types of roaming dynamics apparently behave differently.

Formic acid (HCOOH) belongs to the family of carbonyl compounds, and its photodissociation dynamics has been popularly investigated for more than two decades^28–43^. When its electronic band S_1_ in the range 220 ∼ 250 nm is excited, the dissociation channel mainly contains HCO + OH with a quantum yield of 0.7∼0.8^30^. Because the excited surface is dissociative, the produced fragments bear very large translational energy^30,33^. Kim and co-workers^33^ photolyzed the HCOOH molecule at 193 nm, and obtained a fraction of available energy into translational energy of OH as large as 0.82, whereas the fraction into internal energy of HCO was only 0.13. The fragments are mainly from the decomposition along the S_1_ surface and the resulting product energy disposals are consistent with those obtained at 225 nm^29^.

In the UV photodissociation of formic acid, two molecular channels are found:

1 $${\rm{HCOOH}}+{\rm{h}}v\to {\rm{CO}}+{{\rm{H}}}_{2}{\rm{O}}$$

2 $${\rm{HCOOH}}+{\rm{h}}v\to {{\rm{CO}}}_{2}+{{\rm{H}}}_{2}$$

Given photolysis of cis-formic acid at 193 nm, Khriachtchev *et al*.^35^ observed a CO/CO_2_ product ratio of 0.4 in an Ar-matrix absorption experiment, and a ratio of 5 when trans-formic acid was substituted. Such a CO/CO_2_ ratio is associated with conformational memory which is used to evaluate the extent of isomerization conversion between cis- and trans-formic acid. In contrast, Su *et al*.^34^ obtained the CO/CO_2_ ratio of 11 in the trans-formic acid at 193 nm by using 18 μs-resolved Fourier-transform infrared (FTIR) emission spectroscopy. In the quasiclassical trajectory calculations, Martınez-Nunez group^39^ evaluated a CO/CO_2_ ratio of 22.3 and 12.2 for trans- and cis-HCOOH, respectively, thus corresponding to a ratio of ~2 for trans-/cis-state selected in the initial excitation, which is much smaller than the absorption result of 12.5. Apart from a significant role of the Ar-matrix effect, Morokuma and co-workers^42^ recently suggested that the variation of CO/CO_2_ ratio should be closely related with the following dissociation process, in which the excited HCOOH molecules first flow through S_2_/S_1_ conical intersection and the partial dissociation H·····HCOO is followed by H-roaming on the S_1_ PES to form H_2_ + CO_2_. It is apparently controversial concerning a reliable evaluation of conformational memory which depends on the extent of energy randomization along the photodissociation coordinates.

The roaming pathways in photodissociation of formic acid at 193 nm were recognized theoretically a decade ago^42,43^. In addition to the H-roaming to form H_2_ + CO_2_ on the S_1_ state^42^, when the HCOOH molecules flow through the S_1_/S_0_ conical intersection, the molecular channel CO + H_2_O may be produced via the OH-roaming dynamics on the S_0_ state. The roaming mechanism has been examined in the recent work by Wang group^44^. They detected the CO(v = 0, J ≤ 20) ion imaging in photodissociation of HCOOH at 230 nm using (2 + 1) resonance-enhanced multiphoton ionization (REMPI) technique, and characterized the roaming/TS behavior for CO translational bimodality. However, it is still lacking a deep understanding of the energy disposal in CO(v ≥ 1, J) and discerning of the energy distributions between CO and H_2_O roaming products.

To complement the roaming results by ion imaging, this work employed time-resolved FTIR emission spectroscopy to monitor the fragments in photodissociation of trans-HCOOH at 193 nm. The ensuing FTIR spectra revealed simultaneously two molecular channels of CO + H_2_O and CO_2_ + H_2_. The vibrational state populations of CO (v = 1–8) accompanied by the corresponding rotational energy distributions are analyzed with the aid of spectral simulation. In addition, the ro-vibrational profile of H_2_O roaming co-product is simulated to understand the interplay with the CO product, especially in internal energy partitioning. Finally, the temporal dependence of CO/CO_2_ (v ≥ 1) ratio is obtained to deeply characterize the conformational memory and further reconcile with the previous reports which are in conflict^34,35,39^.

## Results and Discussion

### CO(v = 1) bimodal rotational distribution

A time-resolve FTIR spectrum with 10 cm^−1^ spectral resolution and 5 μs temporal resolution obtained the CO and CO_2_ in the region of 2000–2400 cm^−1^, in which the CO_2_ product (about 2200–2400 cm^−1^) relaxed rapidly within 20 μs delay time (Fig. S1). In addition, some weak signals appeared in the region of 2500–5000 cm^−1^ (Fig. S2). The spectra around 4000 cm^−1^ is ascribed to the CO overtones (Δv ≥ −2), while the region around 2950 cm^−1^ may be due to C-H stretching of the HCOO moiety in the H + HCOO channel^45^. However, the (v_1_,v_2_,v_3_ = 0,0,1) → (0,0,0) vibrational emission of H_2_O molecule fails to appear in the region 3600–3800 cm^−1^.

**Figure 1:**
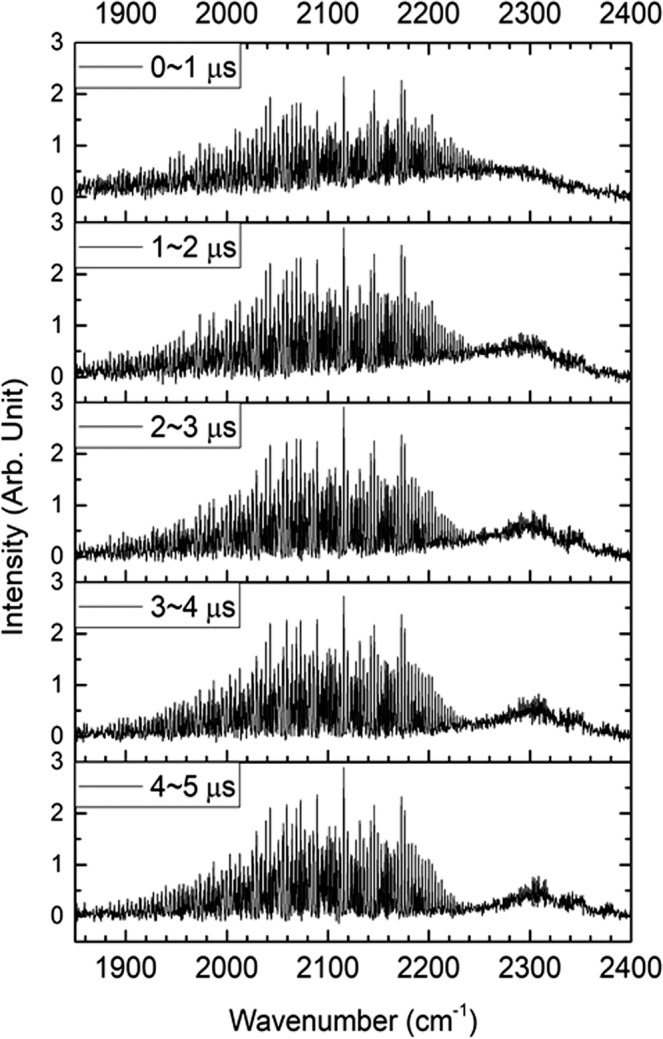
Time-resolved CO and CO_2_ emission spectra (1850–2400 cm^−1^) with a spectral resolution of 0.5 cm^−1^ in photolysis of HCOOH at 193 nm in the presence of Ar at 4 Torr. The CO(v) rotational lines spread from 1880 to 2240 cm^−1^ lying above a large portion of the CO_2_ lines within the initial 2 μs interval. Then, the blue-shift of CO_2_ band with time is indicative of rapid ro-vibrational relaxation.

When the spectral resolution was increased to 0.5 cm^−1^, the CO and CO_2_ spectra with a 1 μs temporal resolution are shown in Fig. 1. The CO spectral intensity, spreading in the region of 1900–2220 cm^−1^, gradually increased with time within the initial 5 μs period, whereas the CO_2_ spectra with a peak at 2300 cm^−1^ were observed to shift to the larger wavenumber, indicating a rapid relaxation from higher vibrational states to lower states. Further, as the delay time is prolonged up to 25 μs (Fig. S1), both the CO and CO_2_ emission intensities reach the maximum at 5–10 μs delay and then the CO_2_ intensities diminish more rapidly. The CO and CO_2_ spectra show distinctly different temporal behavior.

**Figure 2:**
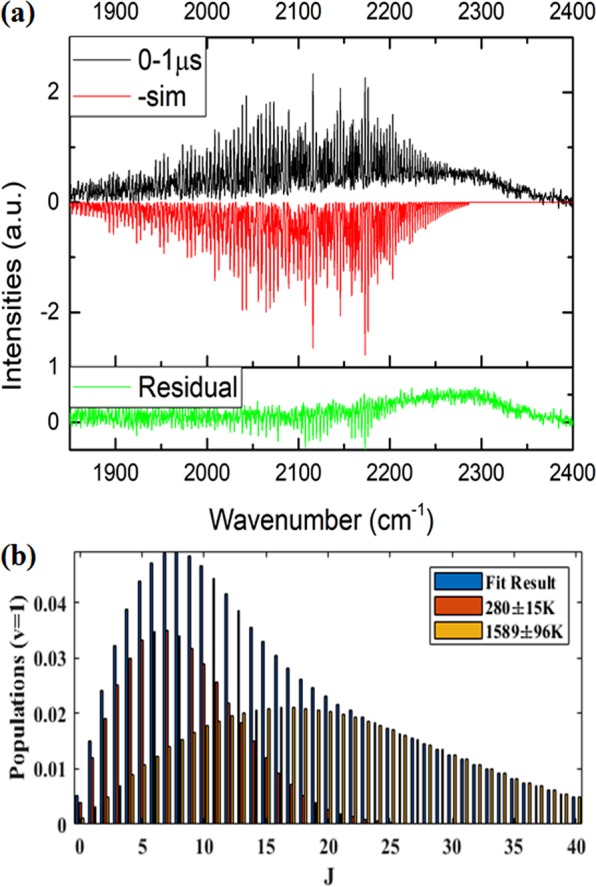
(**a**) The CO and CO_2_ emission spectra (1850–2400 cm^−1^) within the 0–1 μs interval and the counterpart of the CO spectral simulation which ignores contribution of the CO_2_ spectrum. The residue between their spectral difference is also given. (**b**) The rotational distribution simulated at v = 1 breaks up with two Boltzmann rotational components; the small, lower rotational profile with area of 0.41 is ascribed to roaming dynamics, while the other large, higher one with an area of 0.59 is due to the transition state pathway.

As restricted to the spectral congestion, a spectral simulation of the CO population was used to resolve the rotational lines J up to 50 for each vibrational state v from 1 to 8, as shown in Fig. 2a. For lower vibrational states, the profile was found to comprise two Boltzmann rotational components. For instance, while inspecting the rotational population at v = 1 with a 1 μs resolution, a bimodal profile composed of two Boltzmann distributions was obtained with a small low-rotational component, which was ascribed to the OH-roaming on the S_0_ surface, and the other large high-rotational component ascribed to the TS pathway to form the same CO + H_2_O products (Fig. 2b). The CO low- and high-rotational temperatures were evaluated to be 280±15 and 1589±96 K at 0–1 µs delay. A series of rotational temperatures were similarly evaluated for 1–2, 2–3, 3–4, 4–5 and 5–6 µs delay time. Then, the temperatures were extrapolated to the 0 μs delay to be free from the Ar collision, thus obtaining 334 ± 153 and 1344 ± 430 K for both low- and high-rotational components. The evaluated ratio of low/high component varies slightly from 0.41 to 0.23 within 1 to 6 μs interval and is then averaged to 0.26 for v = 1 (Fig. S3).

According to the ion imaging results^44^, a clear bimodal translational distribution appears in the CO (v = 0, J = 9), in which the small component has a low speed peaking at ~380 m/s, in contrast to a large component peaking at ~1400 m/s. When the rotational level increases to J = 20, the low speed component diminishes to almost the minimum. Accordingly, the CO roaming product is characteristic of low rotation accompanied by small translational energy, while CO via the TS pathway is produced in the large rotational levels carrying a large translational energy. Alternatively, while inspecting the transition state structures of TS and roaming pathways on the S_0_ surface^42^, the distance between O and C in the HO···CHO intermediate was evaluated to be 1.865 and 3.005 Å, respectively. Therefore, a shorter O ··· C distance may exert a stronger torque to induce a larger rotational energy carried by the CO product via the TS pathway.

Note that the CO produced in triple fragmentation (H + CO + OH) may be neglected, because the fraction of available energy partitioning into the internal energy of HCO is 0.13^33^. The internal energy obtained by HCO is about 22 kJ/mol, which is close to the dissociation energy threshold of H···CO (~5300 cm^−1^)^44,46,47^. While taking into account the energy partitioning into rotational degrees of freedom and other vibrational modes, the reaction HCO → H + CO becomes negligible. It might be the reason why this channel has never been mentioned in the theoretical evaluation^39,42,43^.

### CO(v = 1–8) bimodal vibrational distribution

**Figure 3:**
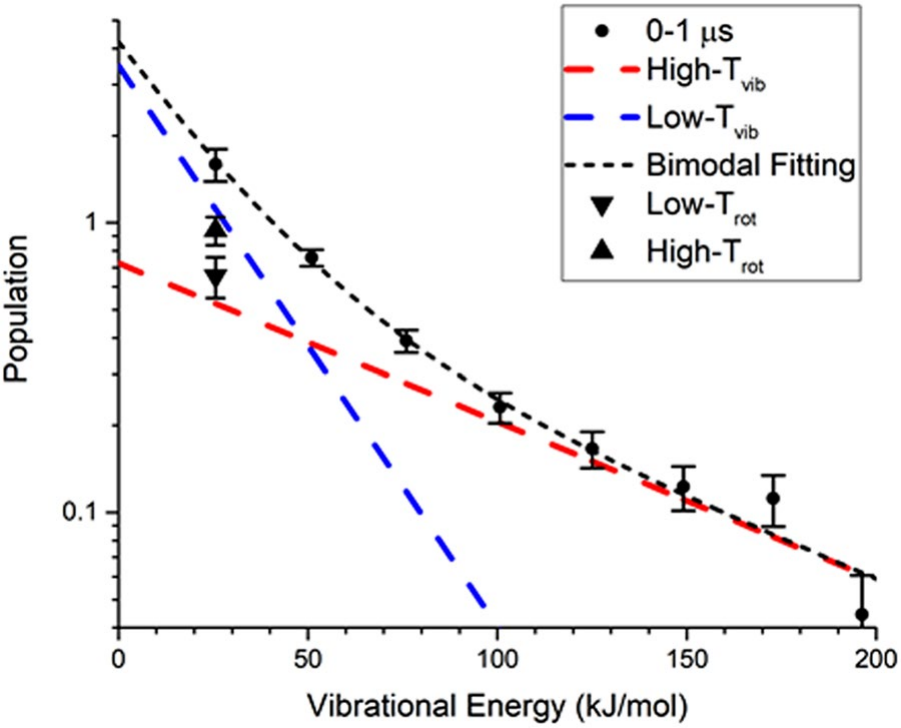
Semi-logarithmic plot of bimodal vibrational population as a function of vibrational energy corresponding to each vibrational state within the 0–1 μs interval. Dotted lines indicate a fit of the vibrational bimodality, while dashed lines correspond to each Boltzmann vibrational component based on Eq. (3). ▲ and ▼ symbols represent the rotational population corresponding to the high and low T_rot_ at v = 1, respectively. Error bars denote an uncertainty of 1σ.

**Figure 4:**
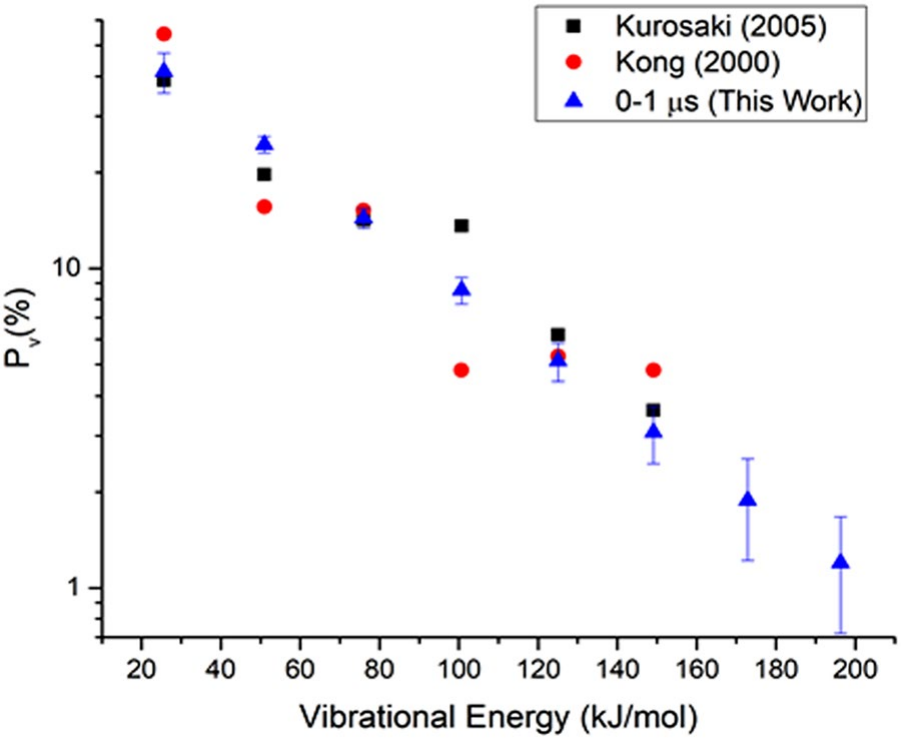
Comparison of a fraction of vibrational population as a function of vibrational energy. The sum of all the vibrational populations is used as denominator to evaluate each vibrational fraction. Our results are essentially consistent with the data (v = 1–6) from Kong and Kurosaki groups. Error bars in this work correspond to 1σ.

The vibrational population of a given state from v = 1 to 8 was evaluated by summing each rotational line simulated up to J = 50. Figure 3 shows the vibrational intensity as a function of vibrational energy distribution, corresponding to v = 1–8, but the zero-point energy is not included. Given the sum of vibrational population as denominator, the corresponding fraction of vibrational population (v = 1–8) appears consistent with the previous results (v = 1–6)^34,38^, as show in Fig. 4. Note that the vibrational fraction at v = 1 obtained by Su *et al*.^34^ is larger than either this work or theoretical calculation by Kurosaki *et al*.^38^. It might arise from partial vibrational relaxation to lower states during data acquisition by the FTIR apparatus with a prolonged temporal resolution^34^.

Each data point of vibrational intensity *I(v)* may break up with two Boltzmann vibrational profiles as expressed by,

3 $$I(v)=A{e}^{{E}_{v}/k{T}_{1}}+B{e}^{{E}_{v}/k{T}_{2}}$$

where A and B are weighing factors, *E*_*v*_ the vibrational energy at v state, *k* the Boltzmann constant, and *T*_1_ and *T*_2_ are the vibrational temperature at different component. To optimize the parameters A, B, T_1_ and T_2,_ two slopes are plotted, yielding a low vibrational and a high vibrational temperature. Their intercepts indicate the factor of A and B. Since the vibrational energy transfer is much slower than the rotational energy transfer, the low and high vibrational temperatures remain constant within the first 6 μs period, corresponding to an average of 2700 ± 70 K and 7980 ± 220 K (Fig. S4). While inspecting the structures calculated for TS and OH-roaming saddle points^42^, the CO bond distance is 1.164 and 1.196 Å, respectively. Despite a slight difference of bond distance, we expect the lower vibrational temperature is ascribed to the TS pathway, while the higher temperature is caused by the roaming route. It is for the first time to find out vibrational-state dependence of the roaming signature.

**Figure 5:**
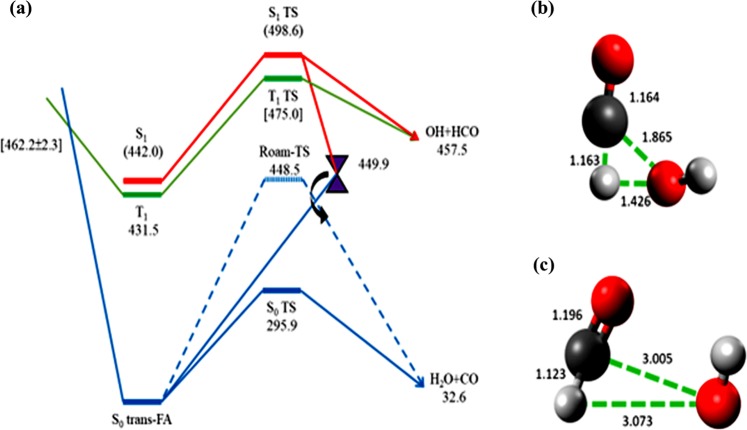
(**a**) Partial potential energy diagram for decomposition of formic acid calculated by Morokuma group (*J Phys Chem Lett* **3**, 1900–1907 (2012)). The singlet state S_0_ is denoted in blue, S_1_ in red, and triplet state T_1_ in green. S_1_/S_0_ conical intersection is denoted as purple cone, while the energy state at 462 kJ/mole denotes the minimal energy of seam crossing. Transition state structures of TS and roaming pathways on the S_0_ potential energy surface are displayed in (**b**,**c**), respectively. The unit of the bond distance are in Angstrom (Å).

Further, according to the theoretical PESs calculations as referred to Fig. 5 ^42,43^, the molecules excited at 193 nm have to proceed through S_1_/T_1_ (431.5~442.0 kJ/mol) and T_1_/S_0_ (462.2 ± 2.3 kJ/mol) ISC to reach the ground state with enough energy to surpass the barrier to form CO + H_2_O. The rate for this route should be slowed by the multi-ISC processes such that the available energy carried may readily relax or disperse. In contrast, the excited molecules concomitantly proceed through the S_1_/S_0_ (449.9 kJ/mol) conical intersection to partially dissociate to HCO···OH in which the OH-roaming may form the same molecular products on the S_0_ surface. As such, the molecular channel via TS mechanism is anticipated to be slower with less internal energy partition than the molecular production via roaming pathway. In addition, upon irradiation of 193 nm, the C=O bond in HCOOH is elongated significantly. Following dissociation to form HCO and OH radicals, most internal energy carried by HCO is favorably deposited in the CO vibrational stretching^40^. The OH-roaming around the HCO core thus results in CO which bears large vibrational energy. The CO roaming channel should prevail over the TS pathway especially for higher vibrational states. On the contrary, at lower vibrational states such as v = 1, the Boltzmann rotational component of CO via the TS pathway becomes larger than the Boltzmann component via the roaming dynamics, as shown in Fig. 3.

Such roaming results exhibit a consistent trend as photolysis of propionaldehyde (CH_3_CH_2_CHO) leading to CO + C_2_H_6_ molecular products, in which the CO roaming behavior was theoretically recognized to be rotational cold but vibrational hot^27^. Nevertheless, these two roaming types are different from each other. Instead of proceeding through a conical intersection, the photodissociation process of propionaldehyde has to surpass a loose roaming saddle point which shows one small imaginary frequency along with some small frequency modes. Two radical moieties HCO and C_2_H_5_ in the roaming saddle point are weakly bound to each other such that they may have chance to roam around varied configurations prior to abstraction reaction.

### Ro-vibrational energy profile of H_2_O co-product

In the photodissociation of trans-HCOOH at 193 nm, a partial portion populated to the S_2_ state led to the HCOO + H products^42^, in which a small amount of HCOO was identified in the IR spectrum around 2950 cm^−1^. Most population dissociated rapidly along the S_1_ repulsive surface to the OH + HCO radical channel. In addition, a small portion flowed through the S_1_/S_0_ conical intersection to form the CO + H_2_O products via the OH-roaming pathways^42^. The feature of CO roaming product has been recognized to possess cold rotational but hot vibrational states.

**Figure 6:**
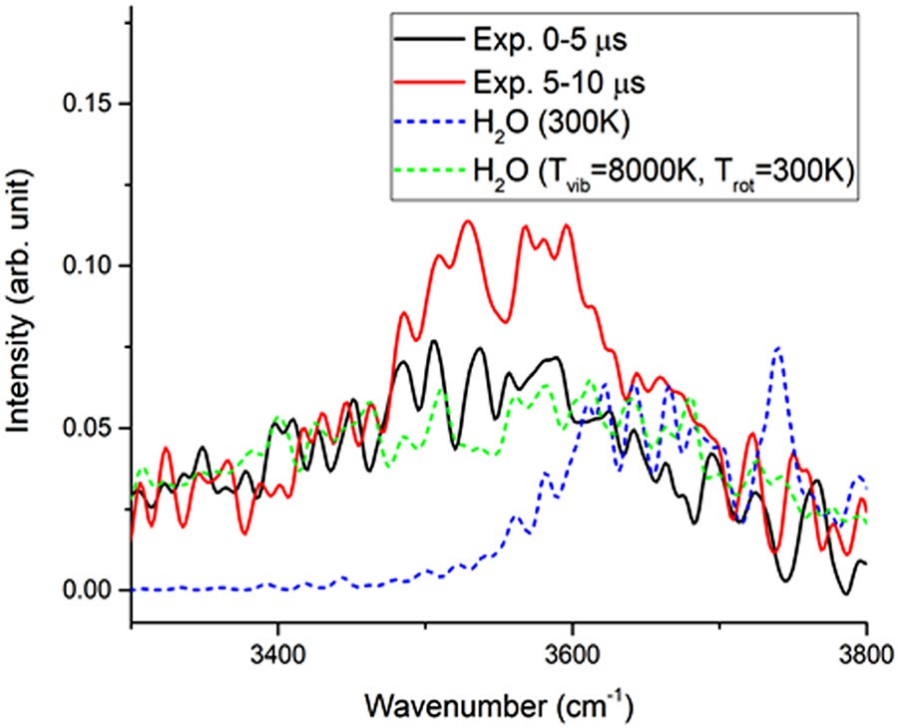
H_2_O spectra in the range of 3300–3800 cm^−1^. The experimental spectra with a spectral resolution of 10 cm^−1^ are indicated in black for 0–5 μs and in red for 5–10 μs. The simulated counterpart denoted in green dashed line was calculated under the condition of vibrational temperature of 8000 K and rotational temperature of 300 K. In comparison, a second simulated counterpart in blue dashed line was calculated with the vibrational temperature of 300 K.

As predicted in the roaming mechanism, the H_2_O co-product is expected to be vibrationally hot, but has not been examined yet. When OH roams around the HCO core followed by an H-atom abstraction at a long distance, the resulting H-OH should be vibrationally excited. Further, given transition lines, Einstein emission coefficients, harmonic wavenumber and anharmonicity constants for calculation of ro-vibrational intensities and energies^48,49^, the H_2_O spectrum was simulated in the range of 3300–3800 cm^−1^, while assumed to have vibrational temperature 8000 K and rotational temperature 300 K, as shown in Fig. 6, which is satisfactorily consistent with the experimental findings, especially in the 0–5 µs delay. The simulated counterpart has included the vibrational modes of symmetric stretching v_1_ = 5, bending v_2_ = 15 and asymmetric stretching v_3_ = 7. Given the emission transition only at (v_1_,v_2_,v_3_) = (0,0,1) → (0,0,0), the H_2_O spectrum simulated at 300 K is blue-shifted by >300 cm^−1^ to the 3600–3900 cm^−1^ region, while compared with the vibrationally excited water. That might be the reason why Su *et al*. could not identify the H_2_O production with similar apparatus^34^.

Note that the H_2_O product via the TS pathway is expected to lie in low vibrational states. The lack of a significant bimodal ro-vibrational distribution in Fig. 6 might rise from the fact that the low-temperature ro-vibrational component was buried in the high-temperature component. In addition, different from the CO product, the multi-atomic H_2_O co-product has more complicated spectra which cannot be spectrally resolved under our experimental conditions, and thus discerning ro-vibrational bimodality is more difficult.

In this work, we have characterized the internal energy distributions between the CO and H_2_O roaming products. To the best of our knowledge, this is the first case to spectrally simulate a multi-atomic co-product in the roaming dynamics. In comparison, for the photodissociation of CH_3_CHO → CO + CH_4_, the CO roaming product has been detected by ion imaging and time-resolved FTIR emission spectroscopy^1,6^, whereas the CH_4_ roaming co-product was detected only by the latter method and then analyzed by the principle of maximum entropy^6^, instead of the spectral simulation.

### Temporal dependence of conformational memory

When cis- or trans-HCOOH is initially excited at 193 nm, if the molecular decomposition is faster than the randomization of the torsion coordinate mixing of the conformers, then the conformational memory may be observed in terms of the CO/CO_2_ ratio which depends on the initial conformer to be excited^35^. Martınez-Nunez *et al*.^39^ ascribed a smaller (CO/CO_2_)_trans_/(CO/CO_2_)_cis_ evaluated of 2 to the difference in funneling geometries of S_1_/S_0_ between cis and trans isomers and Ar-matrix effect in the experiment. They expected more than 30% of the H_2_ + CO_2_ reactive trajectories in the cis isomer funneled through the S_1_/S_0_ conical intersection and then the trajectories rapidly dissociated to the H_2_ + CO_2_ products without any chance to redistribute the energy on the ground state. Morokuma and co-workers^42^ explained conformational memory for a smaller (CO/CO_2_)_cis_ ratio by finding a H-roaming on the S_1_ state to form additional H_2_ + CO_2_ products.

**Figure 7:**
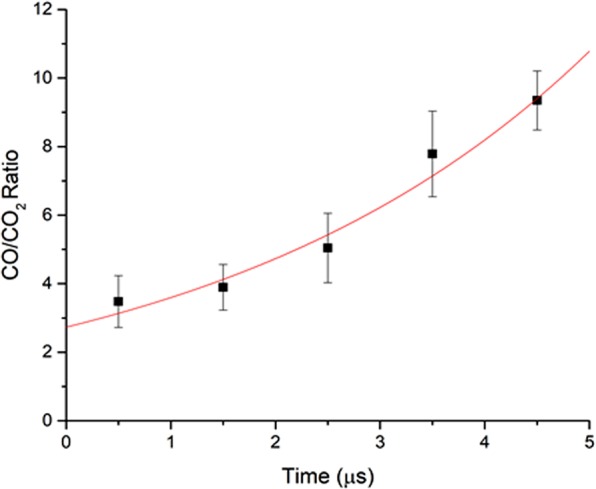
Temporal dependence (0–5 μs) of the CO(v ≥ 1)/CO_2_(v_3_ ≥ 1) ratio in photolysis of trans-HCOOH at 193 nm. Error bars are estimated from the uncertainties of CO_2_ spectra. A single exponential function happens to fit the data points.

Given the Einstein emission coefficients of CO and CO_2_, the integrated area of the high-resolution CO and CO_2_ spectra (Fig. 1) yields a temporal dependence of CO/CO_2_ population ratio. The CO area is evaluated by summing all the rotational lines which sit above the CO_2_ profile. The CO spectra are easily separated from the CO_2_ profile within a small uncertainty, because the CO_2_ profile has weak spectral intensities and poor resolution which is made by the complicated multi-atomic spectra. As shown in Fig. 7, the ratio increases from 3 at 0–1 μs delay time to 10 at 5–6 μs delay, which is consistent with the value of 11 obtained by Su *et al*. using a similar apparatus with a 18 μs temporal resolution^34^. This fact lends the support to a negligible isomerization conversion between cis- and trans-HCOOH by collisions with Ar, which helps enhance the emission signals of the photofragments (see the details in Method). Further, the energy transfer between translational and vibrational degree of freedom is very inefficient, requiring about 10^4^–10^7^ collisions^50^. Thus, Ar-collision effect on the ro-vibrational energy redistribution and isomerization probability of excited state formic acid is minimized. This work provides a detailed temporal behavior of the conformational memory, thus characterizing the early time evolution of the CO and CO_2_ products in two molecular channels.

As mentioned above, (CO/CO_2_)_trans_ yielded a ratio of 5 in the Ar-matrix absorption experiment^35^. In their absorption experiments, the detected population of CO and CO_2_ were substantially quenched to the ground state with a little amount at excited states. Since the production of the CO + H_2_O molecular channel dominates the CO_2_ + H_2_ products decomposed from trans-HCOOH at 193 nm, the (CO/CO_2_)_trans_ ratio at v ≥ 1 by the emission method is expected to be ≤5. Such an emission result lacks to consider the ground state population. Given the (CO/CO_2_)_trans_ ratio of 11 at v ≥ 1 reported by emission spectroscopy^34^, it is apparently in conflict with the ratio of 5 obtained at v ≥ 0 (or v = 0) by absorption spectroscopy. In comparison, a smaller nascent ratio of 3 for the (CO/CO_2_)_trans_ at v ≥ 1 obtained in this work suggests that the CO/CO_2_ ratio at v = 0 should be larger than one which is consistent with the absorption results. As the delay time is prolonged to 6 µs, the CO emission intensity gradually increases, whereas the CO_2_ emission signal is quenched rapidly such that more population of CO_2_ may relax to the ground state, as shown in Fig. S1 and Fig. 1. That is why the (CO/CO_2_)_trans_ ratio increases with increasing the delay time, as shown in Fig. 7. Thus, the temporal behavior of conformational memory plays a significant role to reconcile with the disputed data reported previously by both absorption and emission spectroscopy.

## Conclusion

By taking advantage of 1 μs-resolved FTIR emission spectroscopy, we have visualized concomitantly two molecular channels of CO + H_2_O and CO_2_ + H_2_, and further characterized the internal energy disposal in both CO and H_2_O roaming products, following photodissociation of trans-HCOOH at 193 nm.

The addition of Ar in the chamber does facilitate the collision-induced internal conversion (IC) and intersystem crossing (ISC) processes to enhance substantially the emission intensities of photofragments. With the aid of spectral simulation, the CO rotational and vibrational distributions each contain two Boltzmann profiles; one is ascribed to the roaming route and the other is due to the TS pathway. The CO roaming product is confirmed to possess the states with cold rotation but hot vibration, while the H_2_O roaming co-product is vibrationally excited.

This work has successfully resolved the puzzle concerning the energy correlation, roaming temperature, and internal state distributions for the CO and H_2_O products via the roaming route. Further, the obtained temporal behavior of conformational memory may well interpret the disputed results reported with different methods.

## Methods

Time-resolved FTIR emission spectroscopy^51–55^ with some modification was employed in this work, as illustrated in Fig. S5. A spectrometer (Bruker, IFS 66 v/s) allowing for evacuation of background air and moisture was operated in the step-scan mode, with which the movable mirror of the interferometer can be controlled to move step-by-step.

A 10 ns pulsed ArF excimer laser (GAMlaser, Ex 10) with a repetition rate of 23 Hz was used as a photolysis source emitting at 193 nm. The incident beam was optimized to the energy range of 5–8 mJ/pulse prior to focusing with a spherical lens to reduce its beam size from 8 × 4 to approximately 3 × 3 mm^2^. About 80% energy was remained in the center of the cubic chamber with a dimension 10 × 10 × 10 cm^3^. The sample formic acid (Acros, 97%) was used after several freeze-pump-thaw cycles, and a pressure of 1.0 Torr was then injected in the center of a six-way anodized aluminum cube for reaction. Ar (purity > 99.999%) at a pressure of 4.0 Torr added in the chamber may play two roles: first, to prevent the windows from contamination by the photolyzed species, and second, to enhance the rates of collision-induced IC or ISC to increase the emission intensity^55^.

The emission signal was further enhanced twice by a concave mirror (Thorlabs, CM254-050-P01) placed at the opposite side with respect to the propagating emission signal. The emitting signal was then guided to the FTIR instrument with a pair of 1 in. diameter silver-coated off-axis parabolic mirrors (Edmund, #36–589, and Thorlabs, MPD169-P01), which was flushed with the N_2_ gas to prevent spectral interference by the CO_2_ and moisture in the atmosphere.

An appropriate long-pass filter (Spectrogon, LP-4080 nm) was applied to block the unwanted spectral region and to shorten acquisition time. The transmitted signal was monitored with a high-speed InSb detector cooled at 77 K followed by a short-response-time amplifier (Kolmartech, KISDP-1-LJ2/PS). The output was connected to a low-noise preamplifier (SRS, SR560) which further amplified the electric signals by a factor of 10^4^, and then the signals was recorded by a 130 MS/s transient recorder (Spectrum-Instrumentation, M4i-4410-x8). The temporal resolution can be shortened to 7.7 ns. In this work the digitized signals were repeatedly collected for 30 laser shots at a time interval 7.7 ns, and then the following 130 steps were successively moved and the acquired data were summed up. As such, the temporal resolution of obtained spectrum was prolonged to 1 μs. Experiments were repeated under the same conditions; then, the interferograms were co-added over eight times and finally Fourier transformed to give rise to a series of time-resolved spectra.

In comparison with our previous apparatus^51–55^, a 200 KHz 16-bit transient digitizer was used for signal processing, which restricted the temporal resolution of IR signal to 5 μs. Despite a slower IR response, with respect to the optical response, a detailed spectral variation can be visualized even within the initial 5 μs period.

A Mid-IR emitter inside the FTIR, corresponding to temperature of 1275 K when powered, was used as an external light source to calibrate the system before analyzing the acquired time-resolved spectra. Its spectral feature resembled a blackbody radiator. The spectral responses of beam splitter, optical filters, and detection system were all calibrated.

Why may the addition of Ar in the reaction chamber help enhance the emission intensities of the fragments? An example is shown in Fig. S6 comparing the enhancement of emission signal between 1 and 4 Torr Ar addition in the chamber. Under the experimental conditions, 1 Torr of Ar has a density of 3.5 × 10^16^ molecules/cm^3^ and a relative speed of about 5 × 10^4^ cm/s with respect to HCOOH at 300 K. Given the collision cross section of 35 Å^2^, each excited HCOOH molecule may encounter ∼6 collisions with Ar within 1 μs when the fragments are detected. That is why the addition of Ar may facilitate the level-to-level coupling rate between S_1_ and the dramatically increased density of states in S_0_ or T_1_ at higher excitation energy. In this manner, the Ar-collision induced internal conversion (IC) or intersystem crossing (ISC) processes may be enhanced to remain a large available energy in the molecular channel; thus, CO may gain larger vibrational excitation energy. Note that the colliders simultaneously quench the ro-vibrational distributions of the resultant products.

In addition to enhancement of the emission intensities, the shape of the emission profile may change to some extent (Fig. S6). It may rise from the fact that the energy transfer between translational and rotational degree of freedom is fast such that the Ar collisions may readily change the spectral distribution. In addition, the relative intensities are changed during evolution in the earlier stage.

## Supplementary information

Supplementary information.

## Data Availability

All the relevant data are available from the authors upon request.

## References

[1] Houston PL, Kable SH (2006). Photodissociation of acetaldehyde as a second example of the roaming mechanism. Proc. Natl. Acad. Sci. USA.

[2] Rubio-Lago L (2012). Imaging the molecular channel in acetaldehyde photodissociation: roaming and transition state mechanisms. Phys. Chem. Chem. Phys..

[3] Heazlewood BR (2008). Roaming is the dominant mechanism for molecular products in acetaldehyde photodissociation. Proc. Natl. Acad. Sci. USA.

[4] Lee KLK (2014). Two roaming pathways in the photolysis of CH3CHO between 328 and 308 nm. Chem. Sci..

[5] Han YC, Tsai PY, Bowman JM, Lin KC (2017). Photodissociation of CH3CHO at 248 nm: Identification of the channels of roaming, triple fragmentation and the transition state. Phys. Chem. Chem. Phys..

[6] Li HK (2015). Communication: photodissociation of CH3CHO at 308 nm: observation of H-roaming, CH3-roaming, and transition state pathways together along the ground state surface. J. Chem. Phys..

[7] Shepler BC, Braams BJ, Bowman JM (2007). Quasiclassical trajectory calculations of acetaldehyde dissociation on a global potential energy surface indicate significant non-transition state dynamics. J. Phys. Chem. A.

[8] Suits AG (2008). Roaming atoms and radicals: A new mechanism in molecular dissociation. Acc. Chem. Res..

[9] Chambreau SD, Lahankar SA, Suits AG (2006). Correlated v(H(2)) and j(CO) product states from formaldehyde photodissociation: dynamics of molecular elimination. J. Chem. Phys..

[10] Sivaramakrishnan R, Michael JV, Klippenstein SJ (2010). Direct observation of roaming radicals in the thermal decomposition of acetaldehyde. J. Phys. Chem. A.

[11] Mauguiere FA (2015). Phase space structures explain hydrogen atom roaming in formaldehyde decomposition. J. Phys. Chem. Lett..

[12] Goncharov V, Herath N, Suits AG (2008). Roaming dynamics in acetone dissociation. The J. Phys. Chem. A.

[13] Maeda S, Ohno K, Morokuma K (2010). A theoretical study on the photodissociation of acetone: Insight into the slow intersystem crossing and exploration of nonadiabatic pathways to the ground state. J. Phys. Chem. Lett..

[14] Grubb MP, Warter ML, Suits AG, North SW (2010). Evidence of roaming dynamics and multiple channels for molecular elimination in NO3 photolysis. J. Phys. Chem. Lett..

[15] Grubb MP (2012). No straight path: roaming in both ground- and excited-state photolytic channels of NO3–> NO + O2. Science.

[16] Harding LB, Klippenstein SJ (2010). Roaming radical pathways for the decomposition of alkanes. J. Phys. Chem. Lett..

[17] Tsai PY (2014). Roads leading to roam. Role of triple fragmentation and of conical intersections in photochemical reactions: experiments and theory on methyl formate. Phys. Chem. Chem. Phys..

[18] Nakamura M (2015). Dynamical, spectroscopic and computational imaging of bond breaking in photodissociation: roaming and role of conical intersections. Faraday Discuss..

[19] Lin KC (2016). Regulation of nonadiabatic processes in the photolysis of some carbonyl compounds. Phys. Chem. Chem. Phys..

[20] Dey A (2014). Photodissociation dynamics of nitromethane and methyl nitrite by infrared multiphoton dissociation imaging with quasiclassical trajectory calculations: signatures of the roaming pathway. J. Chem. Phys..

[21] Jordan MJ, Kable SH (2012). Chemistry. Roaming reaction pathways along excited states. Science.

[22] Xiao H, Maeda S, Morokuma K (2011). Excited-state roaming dynamics in photolysis of a nitrate radical. J. Phys. Chem. Lett..

[23] Lu Z (2014). Photochemistry. Evidence for direct molecular oxygen production in CO(2) photodissociation. Science.

[24] Lin K-C (2018). Roaming signature in photodissociation of carbonyl compounds. Int. Rev. Phys. Chem..

[25] Chen C (2010). Evidence for vinylidene production in the photodissociation of the allyl radical. J. Phys. Chem. Lett..

[26] Tsai PY, Li HK, Kasai T, Lin KC (2015). Roaming as the dominant mechanism for molecular products in the photodissociation of large aliphatic aldehydes. Phys. Chem. Chem. Phys..

[27] Tsai PY, Hung KC, Li HK, Lin KC (2014). Photodissociation of propionaldehyde at 248 nm: Roaming pathway as an increasingly important role in large aliphatic aldehydes. J. Phys. Chem. Lett..

[28] Ebata T, Fujii A, Amano T, Ito M (1987). Photodissociation of formic acid: internal state distribution of hydroxyl fragment. J. Phys. Chem..

[29] Ebata T, Amano T, Ito M (1989). Photodissociation dynamics of the S1(nπ*) state of formic acid. J. Chem. Phys..

[30] Singleton DL, Paraskevopoulos G, Irwin RS (1990). Laser photolysis of carboxylic acids in the gas phase: direct determination of the hydroxyl quantum yield at 222 nm. J. Phys. Chem..

[31] Brouard, M., Simons, J. P. & Wang, J. X. State-to-state photodissociation dynamics in formic acid. *Faraday Discuss. Chem. Soc*. **91** (1991).

[32] Langford SR, Batten AD, Kono M, Ashfold MNR (1997). Near-UV photodissociation dynamics of formic acid. J. Chem. Soc., Faraday Trans..

[33] Shin SK, Han EJ, Kim HL (1998). Photodissociation dynamics of formic acid at 193 nm. J. Photochem. Photobiol., A.

[34] Su H (2000). Photodissociation of formic acid. J. Chem. Phys..

[35] Khriachtchev L, Pettersson M, Rasanen M (2002). Conformational memory in photodissociation of formic acid. J. Am. Chem. Soc..

[36] He HY, Fang WH (2003). A CASSCF/MR-CI study toward the understanding of wavelength-dependent and geometrically memorized photodissociation of formic acid. J. Am. Chem. Soc..

[37] Borges I, Rocha AB, Martínez-Núñez E, Vázquez S (2005). Theoretical investigations on the vibronic coupling between the electronic states S0 and S1 of formic acid including the photodissociation at 248 nm. Chem. Phys. Lett..

[38] Kurosaki Y, Yokoyama K, Teranishi Y (2005). Direct ab initio molecular dynamics study of the two photodissociation channels of formic acid. Chem. Phys..

[39] Martínez-Núñez E (2005). Photodissociation of formic acid: A trajectory surface hopping study. Chem. Phys. Lett..

[40] Huang C, Zhang C, Yang X (2010). State-selected imaging studies of formic acid photodissociation dynamics. J. Chem. Phys..

[41] Olbert-Majkut A, Ahokas J, Lundell J, Pettersson M (2010). Photolysis of HCOOH monomer and dimer in solid argon: Raman characterization of *in situ* formed molecular complexes. Phys. Chem. Chem. Phys..

[42] Maeda S, Taketsugu T, Morokuma K (2012). Automated exploration of photolytic channels of HCOOH: Conformational memory via excited-state roaming. J. Phys. Chem. Lett..

[43] Maeda S, Taketsugu T, Ohno K, Morokuma K (2015). From roaming atoms to hopping surfaces: mapping out global reaction routes in photochemistry. J. Am. Chem. Soc..

[44] Ma Y (2019). Roaming dynamics in the photodissociation of formic acid at 230 nm. J. Phys. Chem. A.

[45] Burema SR, Lorente N, Bocquet ML (2012). A theoretical rationalization of a total inelastic electron tunneling spectrum: the comparative cases of formate and benzoate on Cu(111). J. Chem. Phys..

[46] de Wit G (2012). Product state and speed distributions in photochemical triple fragmentations. Faraday Discuss..

[47] Lombardi A (2016). Rovibrationally excited molecules on the verge of a triple breakdown: Molecular and roaming mechanisms in the photodecomposition of methyl formate. J. Phys. Chem. A.

[48] Rothman LS (2010). HITEMP, the high-temperature molecular spectroscopic database. J. Quant. Spectrosc. Radiat. Transfer.

[49] Császár AG, Mills IM (1997). Vibrational energy levels of water. Spectrochim. Acta Part A.

[50] Houston, P. L. *Chemical Kinetics and Reaction Dynamics* 289–292 (McGraw-Hill Componies, Inc., New York, 2001).

[51] Liu YT (2010). Photodissociation of gaseous acetyl chloride at 248 nm by time-resolved Fourier-transform infrared spectroscopy: the HCl, CO, and CH2 product channels. J. Phys. Chem. A.

[52] Liu CY (2011). Gas-phase photodissociation of CH3CHBrCOCl at 248 nm: detection of molecular fragments by time-resolved FT-IR spectroscopy. ChemPhysChem.

[53] Yeh YY (2012). Gas-phase photodissociation of CH3COCN at 308 nm by time-resolved Fourier-transform infrared emission spectroscopy. J. Chem. Phys..

[54] Hu EL, Tsai PY, Fan H, Lin KC (2013). Photodissociation of gaseous CH3COSH at 248 nm by time-resolved Fourier-transform infrared emission spectroscopy: observation of three dissociation channels. J. Chem. Phys..

[55] Hung KC, Tsai PY, Li HK, Lin KC (2014). Photodissociation of CH3CHO at 248 nm by time-resolved Fourier-transform infrared emission spectroscopy: verification of roaming and triple fragmentation. J. Chem. Phys..

