# Supplementary material for: Roaming Dynamics and Conformational Memory in Photolysis of Formic Acid at 193 nm Using Time-resolved Fourier-transform Infrared Emission Spectroscopy
Source: Sci Rep. 2020 Mar 16;10:4769. doi: 10.1038/s41598-020-61642-7 (PMC7075954; doi:10.1038/s41598-020-61642-7)
Supplement: Supplementary file 1 — Supplementary information. [file 41598_2020_61642_MOESM1_ESM.pdf]

Supporting Information

Roaming Dynamics and Conformational Memory in Photolysis of Formic Acid at 193 nm Using Time-resolved Fourier-transform Infrared Emission Spectroscopy

Cheng-Jui Tso[1], Toshio Kasai[1,3], and King-Chuen Lin*[1,2]

[1] Department of Chemistry, National Taiwan University, Taipei 10617, Taiwan

[2] Institute of Atomic and Molecular Sciences, Academia Sinica, Taipei 10617, Taiwan

[3] Institute of Scientific and industrial Research, Osaka University, Ibaraki, Osaka 567-0047, Japan

*To whom correspondence should be addressed.

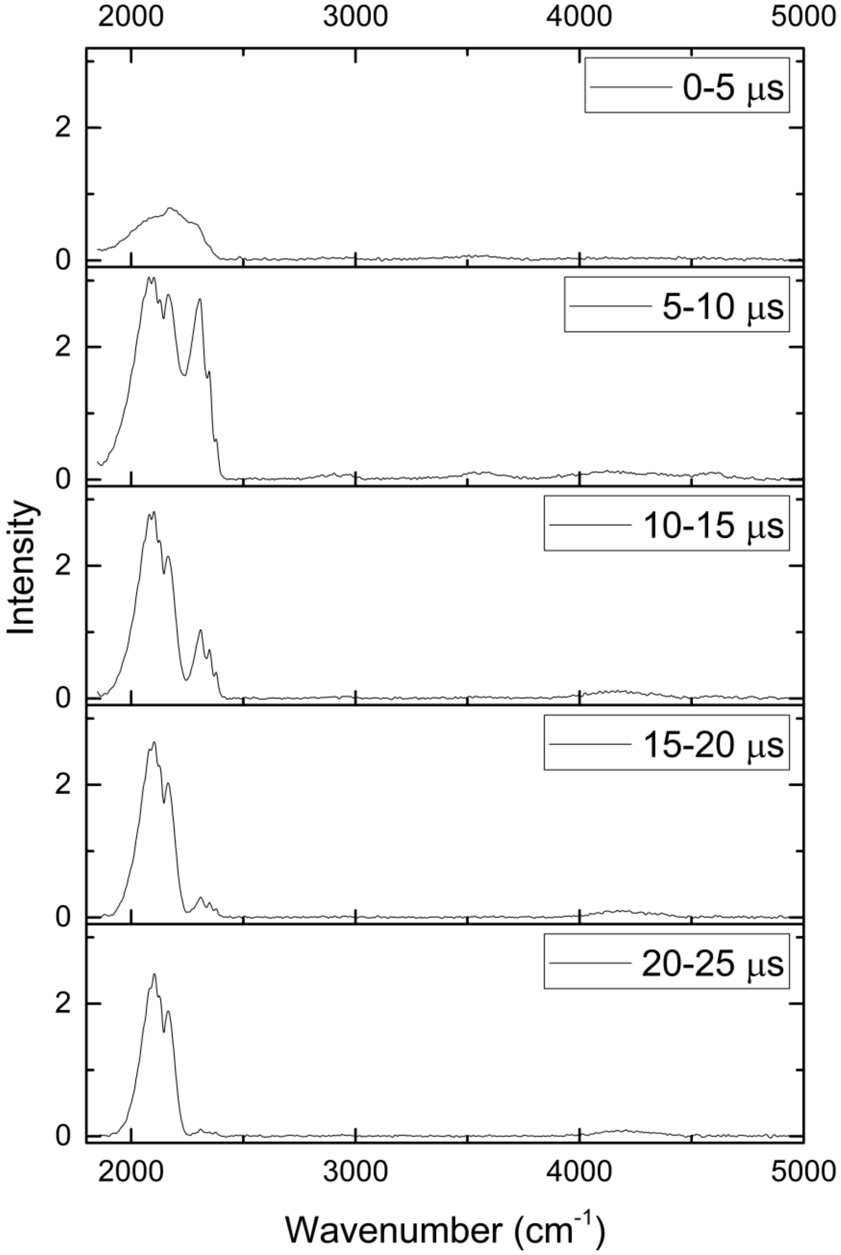

Fig.S1. Time-resolved FTIR emission spectra of the fragments from 1800 to 4500 cm^{-1} with a spectral resolution of 10 cm^{-1} following photolysis of trans-HCOOH at 193 nm. The CO and CO_2 bands at around 2200 and 2400 cm^{-1} originating from the emission transition $\Delta v=-1$ and $\Delta v_3=-1$, respectively. Both CO and CO_2 signals rise with time, but CO_2 relaxes rapidly within 25 μs.

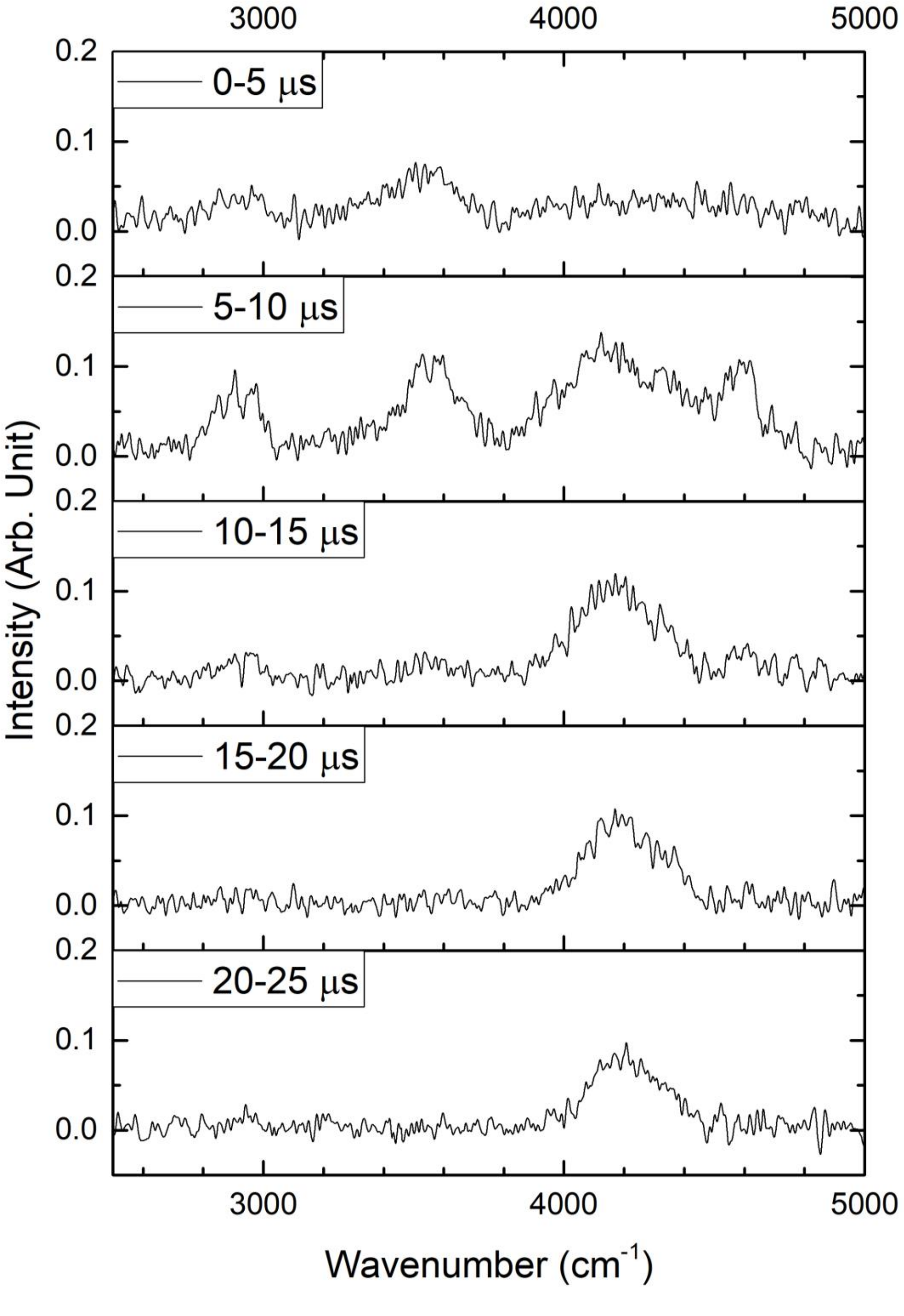

Fig.S2 Time-resolved FTIR spectra of the fragments in the range of 2750-5000 cm^{-1} with a spectral resolution of 10 cm^{-1} after photolysis of formic acid at 193 nm. The band at 4000-4200 cm^{-1} indicate the CO overtones ($\Delta v=-2$) and the bands around 3800 cm^{-1} is due to the highly vibrational H_2O.

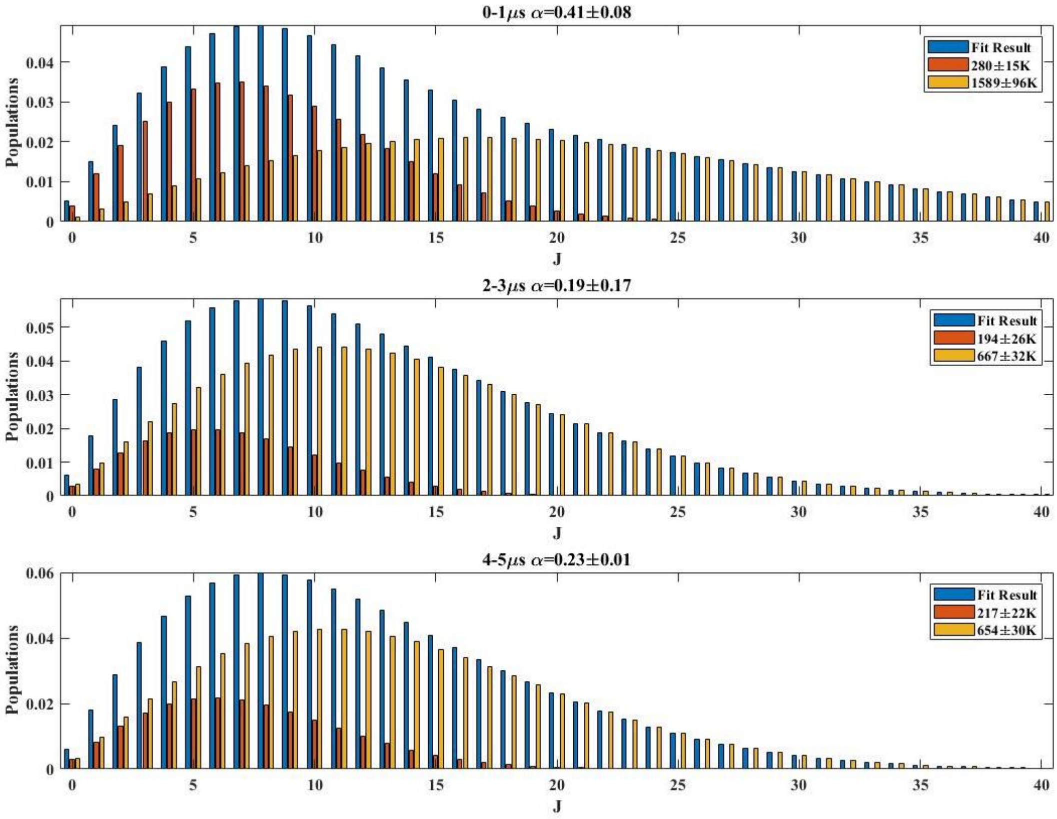

Fig.S3 Rotational distribution at v=1 breaks up with two Boltzmann components ascribed to roaming dynamics and transition state (TS) pathway. The ratio of roaming/TS component evaluated by the individual profile area to be 0.41±0.08, 0.19 ±0.17 and 0.23± 0.01 for the delay time at 0-1, 2-3, and 4-5 μs interval.

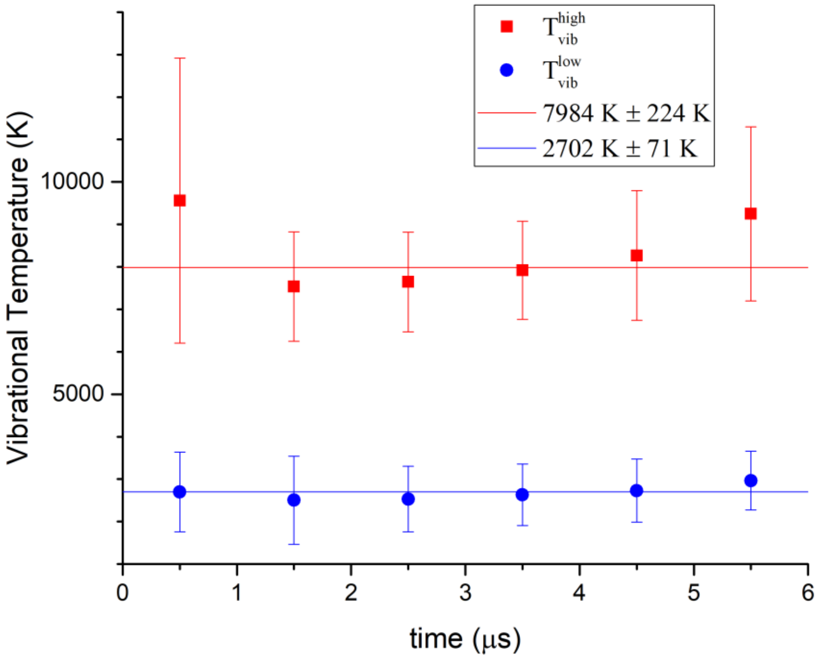

Fig.S4 Temporal dependence (0-6 μs) of low and high vibrational temperature which are averaged to 2702 ± 71 K and 7984 ± 224 K with a 1σ error bar.

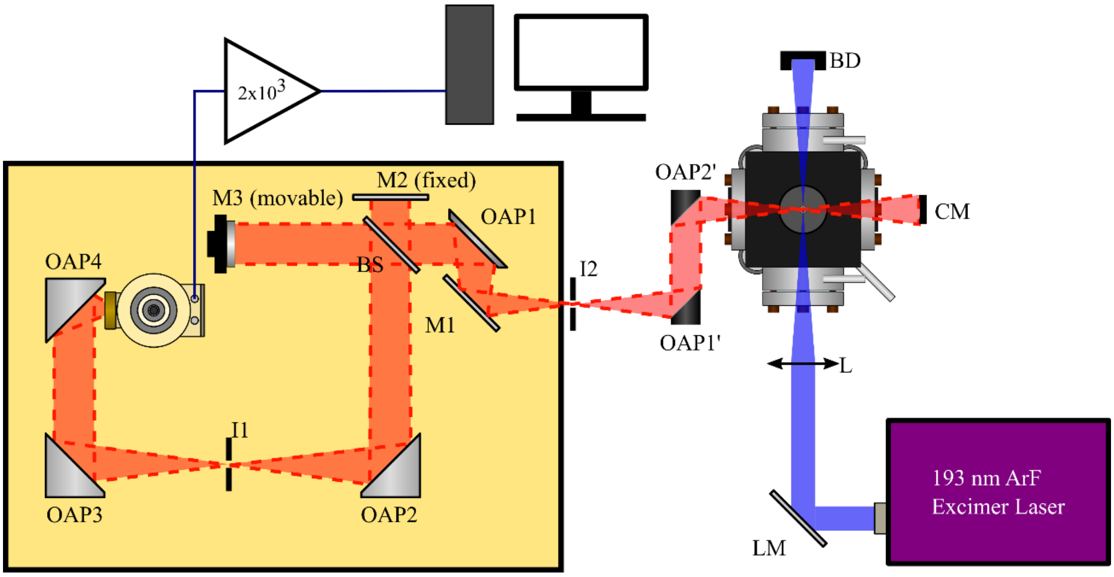

Fig.S5 Schematic diagram of the time-resolved FTIR emission spectroscopy. BD: Beam dumper, BS: Beam splitter, CM: Concave mirror, I: Iris, L: Lens, LM: Laser-line reflective mirror, M: Reflective mirror, OAP: Off-axis parabolic mirror.

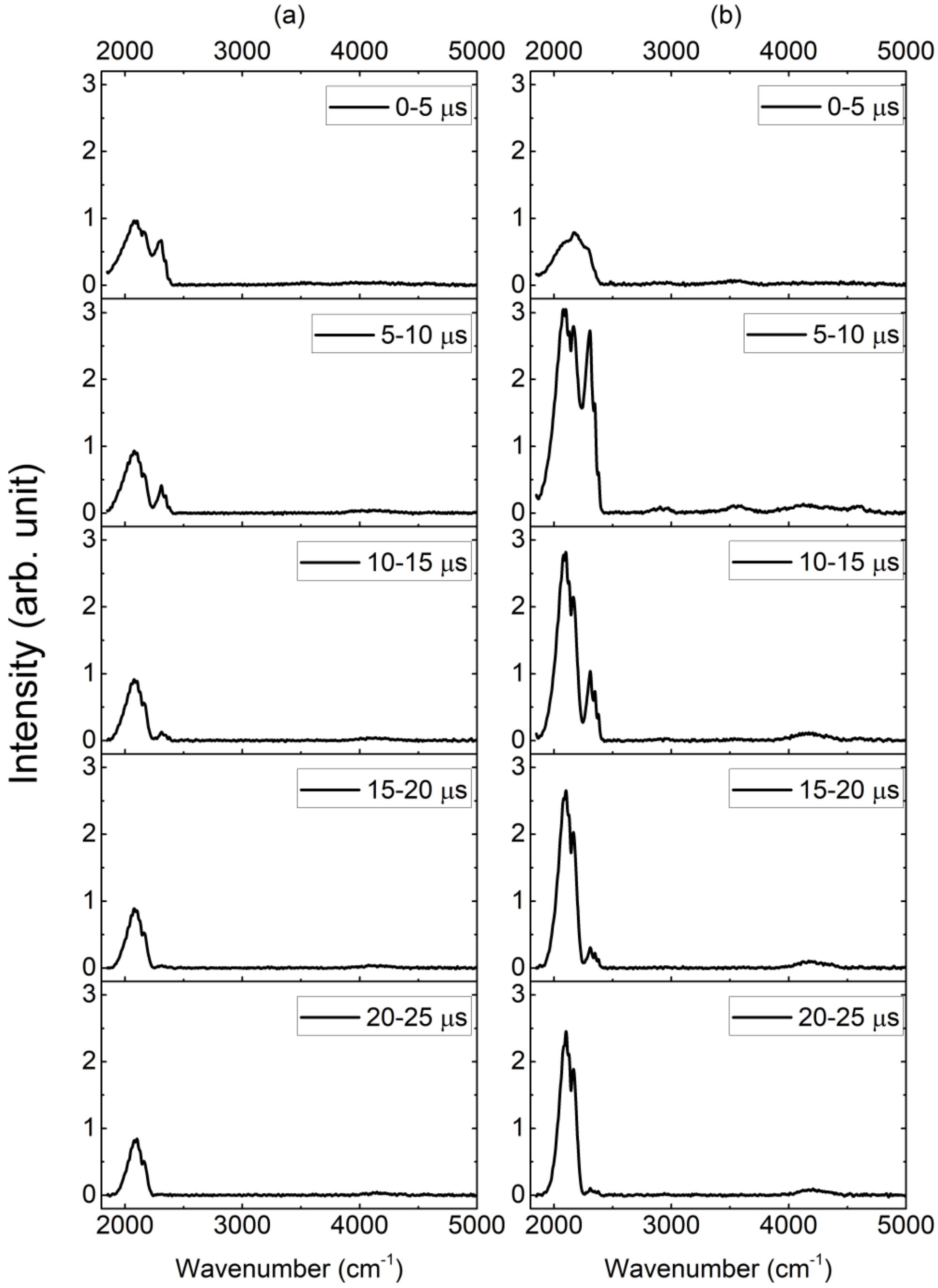

Fig.S6 Time-resolved FTIR spectra of fragments with a spectral resolution of 10 cm^{-1} following photolysis of 1 Torr formic acid at 193 nm, in the presence of (a) 1 Torr Ar and (b) 4 Torr Ar added in the chamber. The strong bands of CO at ~2200 cm^{-1} and CO_2 at ~ 2400 cm^{-1} stem from the Δv=-1and Δv_3=-1 emission transition, respectively. The emission signals are enhanced with increasing the Ar pressure.
